# Case report of an angiosarcoma of the abdominal wall during liraglutide injections: A coincidence?

**DOI:** 10.1016/j.ijscr.2022.107444

**Published:** 2022-07-21

**Authors:** Eric Bergeron, Meriame Dami, Xuan Vien Do, Chantal Vallee, Jonathan Noujaim

**Affiliations:** a Department of Surgery, Charles-LeMoyne Hospital, Greenfield Park, Canada; b Department of Anatomopathology, Charles-LeMoyne Hospital, Greenfield Park, Canada; c Department of Medical Imaging, Charles-LeMoyne Hospital, Greenfield Park, Canada; d Department of Medicine, Charles-LeMoyne Hospital, Greenfield Park, Canada; e Department of Medical Oncology, Maisonneuve-Rosemont Hospital, Montreal, Canada

**Keywords:** Angiosarcoma, Abdominal wall, Liraglutide, Morbid obesity, Case report

## Abstract

• Angiosarcoma is a very rare but highly aggressive malignant vascular tumor.
• Abdominal wall is a very rare site for angiosarcoma and occurs almost exclusively in obese patients.
• Liraglutide injection is explored for a possible association with the occurrence of angiosarcoma of the abdominal wall.

## Abstract (fr)

**Introduction:**

Angiosarcoma is a very rare but highly aggressive malignant vascular tumor. Sporadic cases that develop in the abdominal wall are almost exclusively observed in obese patients. The underlying causes remain unclear. Liraglutide, a glucagon-like peptid-1 agonist receptor treatment, effective for weight management and glycemic control in type 2 diabetes, is not known to be associated with the occurrence of angiosarcoma.

**Case presentation:**

A 62-year-old woman developed a very aggressive, rapidly recurrent angiosarcoma of the abdominal wall. The angiosarcoma developed while she was taking liraglutide injections. The patient died within few months.

**Discussion:**

Angiosarcoma, particularly of the abdominal wall, is very rare, and occurs mainly in obese patients. This cancer had a highly aggressive behavior. The association with liraglutide and angiosarcoma cannot be established nor eliminated from this case and literature review.

**Conclusion:**

Angiosarcoma is a rare and highly aggressive cancer that occasionally originates in the abdominal wall. In this case, in addition to the underlying obesity, a possible association of her liraglutide subcutaneous injections cannot be excluded. In the future, if other cases of abdominal walls angiosarcomas associated with liraglutide subcutaneous injections were to be reported, a possible causality should be further investigated.

## 1 Introduction

Soft tissue sarcomas are a heterogeneous group of cancers that represent <1 % of all adult neoplasms [1]. Angiosarcoma is a very rare but highly aggressive malignant vascular tumor [2], [3], [4], [5] accounting for 1 % to 4 % of all soft tissue sarcomas [2], [3], [4], [5], [6]. Localized angiosarcoma of the abdominal wall is so infrequent that only few anecdotal cases were reported in the literature [3], [7], [8], [9], [10], [11], [12], [13], [14]. Identified risk factors include chronic lymphedema, radiation therapy, chronic sun or chemical toxin exposure, foreign bodies, immunosuppression, and familial syndromes including neurofibromatosis (NF-1), BRCA-1 and BRCA-2 mutations and Klippel-Trenaunay syndromes [2], [5].

We recently managed an obese woman who presented a very aggressive angiosarcoma of the abdominal wall in the context of repeated liraglutide subcutaneous injections, a glucagon-like peptid-1 receptor agonist, effective for weight management and glycemic control in type 2 diabetes [15]. Herein, we present the case questioning a possible causal relationship between liraglutide and angiosarcoma. This work is reported in line with the SCARE criteria [16].

## 2 Case presentation

A 62-year-old woman presented at the emergency room for a progressive mass of the abdominal wall. Up to one month prior to presentation, the patient was injecting herself repeatedly in the abdominal wall with subcutaneous liraglutide for about two years. Except for abdominal liposuction years before, past medical history was irrelevant. The patient had a body mass index of 47. Upon presentation, her vital signs were normal with no history of fever. Physical examination revealed a 20-cm circumscribed mass on the right side of the abdominal wall. It was movable and distinguishable from the muscles of the abdomen. The overlying and surrounding skin showed ecchymoses and “peau d'orange”, but no evidence of cellulitis or wounds.

**Fig. 1:**
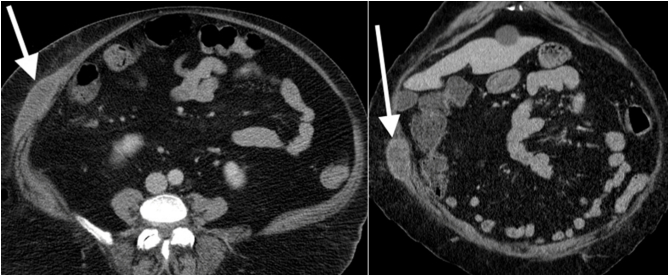
LEFT: Axial contrast-enhanced CT image shows an oblong lesion within the soft tissues of the abdominal wall. RIGHT: Coronal CT image of the same lesion.

A CT scan showed a 9-cm oblong lesion within soft tissues of the abdominal wall. Its structure was complex and composed of isodense and mildly hypodense areas (Fig. 1). There was a slight infiltration of the surrounding fat but no invasion of the fascia and muscles. The initial presumed working diagnosis was a hematoma that occurred because of the repeated subcutaneous injections. She was brought to the operating room to undergo an en-bloc excision of the encapsulated mass. The fascia was free of any pathology. On microscopy, there was no evidence of neoplastic disease since the specimen was entirely composed of blood clots. Therefore, the retained diagnosis was a traumatic hematoma following liraglutide subcutaneous injections. Post-operatively, a vacuum system was left on the open wound. The patient was discharged after three days, and the wound was completely closed after 2 weeks.

**Fig. 2:**
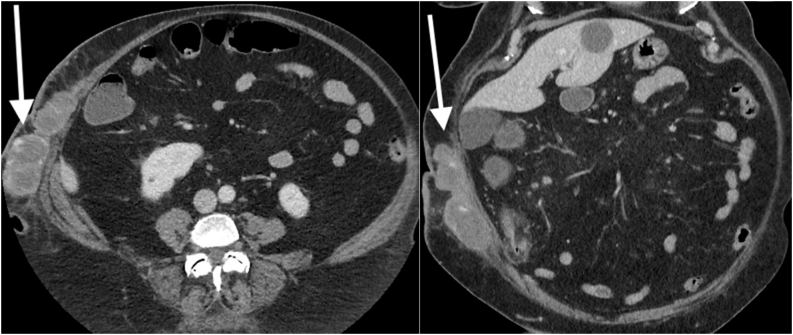
LEFT: Axial contrast-enhanced CT image shows the recurrence of a mass in the same area but is more extensive, lobulated and nodular in appearance. RIGHT: Coronal CT image of the same lesion.

Seven weeks later, the patient presented again at the emergency room complaining that a mass reappeared in the same location. On physical examination, cellulitis of the surrounding skin was evident. The CT scan showed a recurring mass in the same area. However, it was more extensive and had a multi-lobulated, nodular appearance with enhancing walls and vascularized areas (Fig. 2). There was focal thickening of the surrounding cutaneous soft tissue and mild fat stranding. The appearance was very atypical for a hematoma and an underlying neoplastic process was therefore suspected. Intravenous large-spectrum antibiotics were administered. The patient was brought back again to the operating room where a second en-bloc resection of the mass was performed. The gross appearance revealed a mainly hemorrhagic mass. There was still no evidence of neoplastic invasion of the aponeurosis and muscles. A vacuum system was left on the wound. The patient continued to receive antibiotics. She was discharged after 14 days awaiting pathologic analysis.

**Fig. 3:**
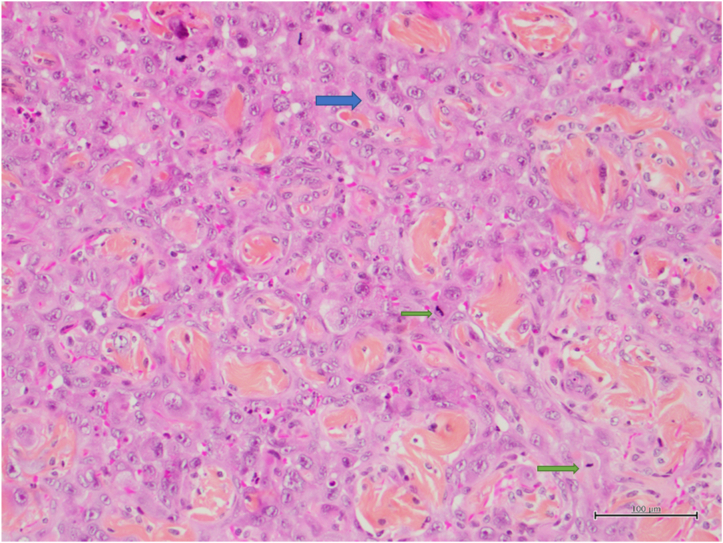
Solid sheets of atypical epithelioid cells with vesicular pleomorphic nuclei and macronucleoli (blue arrow). Note the numerous mitosis (green arrows) (HPS. 10×). (For interpretation of the references to colour in this figure legend, the reader is referred to the web version of this article.)

**Fig. 4:**
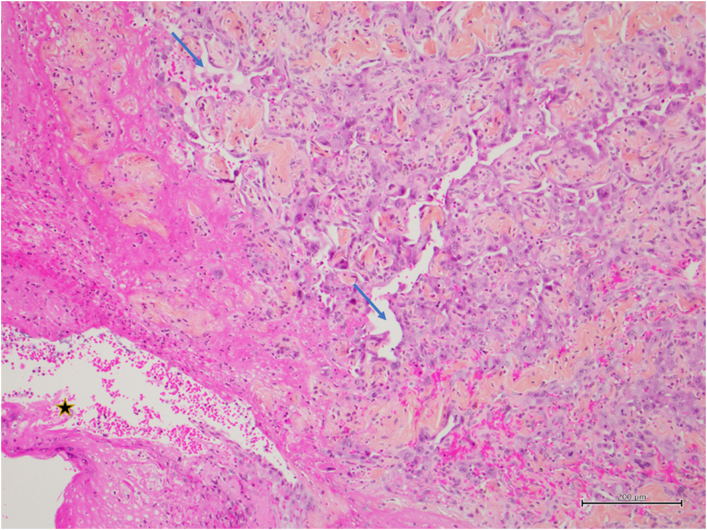
Sinusoid and papillary structures with pseudo-vascular spaces (blue arrow). Note the ulcerated normal epidermis in the lower left corner (black star), (HPS. 10×). (For interpretation of the references to colour in this figure legend, the reader is referred to the web version of this article.)

Sixteen days later, the patient was seen again. Recurrence of the mass was evident. A skin infection was observed once again. New subcutaneous nodules in proximity of the mass were identified. The working diagnosis was now evident as an underlying neoplastic process. The patient was admitted, and antibiotics were prescribed. A third resection was attempted. The specimen revealed again large hemorrhagic abdominal masses. However, upon careful examination, ulcerated subcutaneous hemorrhagic poorly formed tumors could be identified. Microscopic examination revealed high grade epithelioid and pleomorphic cells organized in solid sheets, papillae and anastomotic sinusoid spaces occasionally forming large bloody lakes. The cells were composed of abundant eosinophilic cytoplasm, large vesicular and hyperchromatic nuclei with numerous mitoses. On immunohistochemistry studies, cells expressed CD31 and ERG. The findings were compatible with a diagnosis of high grade epithelioid angiosarcoma (Fig. 3, Fig. 4). Upon reviewing the initial first two resected specimens, foci of angiosarcoma were eventually identified.

**Fig. 5:**
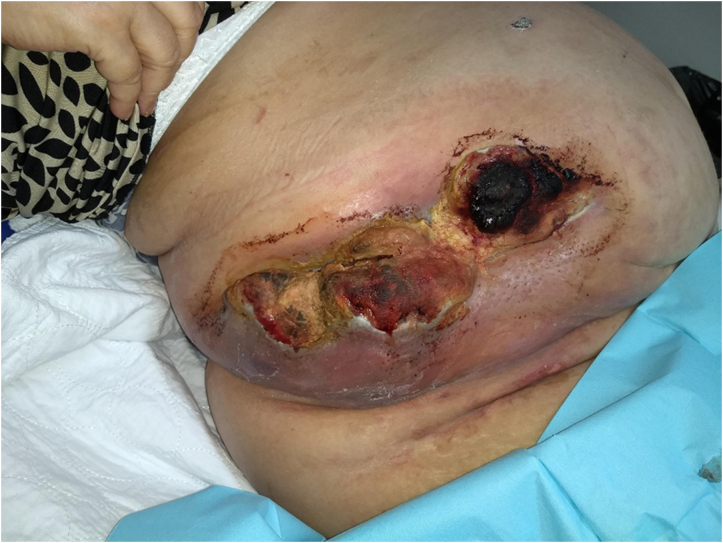
Extensive recurrence of angiosarcoma of the abdominal wall with ulcerations and overt bleeding.

Following the diagnosis of cutaneous angiosarcoma, the patient was transferred to a sarcoma referral center. Upon admission, rapid progression of the tumor was documented; the upper thigh now being involved in addition to the abdominal wall (Fig. 5). A systemic work-up unfortunately showed new onset of metastatic disease with multiple pulmonary metastases and axillary lymph nodes. Furthermore, uncontrollable bleeding was evident in the primary tumor. The patient went on to receive palliative intent radiation therapy (25 Gy in 5 fractions) on the primary site in addition to beginning weekly paclitaxel chemotherapy (3 weeks on, 1 week off). At discharge, the bleeding resolved, and the associated pain was improved. Following 3 cycles of chemotherapy, a slight response of the primary was observed. The patient subsequently experienced new onset of shortness of breath, progressive right thoracic pain and nonsevere hemoptysis. A repeat CT-scan showed a mix response with a decrease in size of previously documented pulmonary metastases, but new hilar lesions presumed to be the source of the bleeding. The patient went on to receive an additional course of palliative radiation therapy which unfortunately was aborted after 4 fractions (20 gy total) because of a positive COVID-19 contact. Her condition continued to deteriorate to eventually succumb eight months after initial presentation from her disease.

## 3 Discussion

Soft tissue sarcomas represent respectively 0.72 % and 0.49 % of all expected cancers in United States [1] and Canada [17]. Angiosarcomas account for only 1–4 % of all soft tissue sarcomas [2], [3], [4], [5], [6]. The cutaneous form of angiosarcomas is the most common, and represents about half of all tumors, with the head and neck being the most frequently involved region [2], [5], [6], [7], followed by the extremities and the breast [7]. Other documented sarcomas of the abdominal wall include undifferentiated pleomorphic sarcomas, fibrosarcomas, and synovial sarcomas [4]. The lower abdominal wall is an exceptional location for angiosarcoma [7], [14], and was only rarely described in case reports and small series [2]. In the largest prospective study, the Memorial Sloan-Kettering Cancer Center group identified 85 cases of soft tissue tumors of the abdominal wall from 1982 and 1999. No case of angiosarcoma was documented [4]. Wang et al. reported a series of 200 cases of angiosarcoma from 2006 to 2014, but here again, not one case involved the abdominal wall [6].

In addition to the previously described risk factors for angiosarcoma, obesity is recurrent finding among reports. [7], [11], [12], [13] Usually, obese patients presenting with cutaneous angiosarcoma have previous typical changes of chronic lymphedema of the abdominal wall (“peau d'orange”) which was present in our patient. Beyond the repeated subcutaneous injections of liraglutide, the patient's obesity certainly remains the main contributing factor. Liraglutide is a glucagon-like peptid-1 (GLP-1) receptor agonist that is a new adjunct in treatment of type 2 diabetes mellitus. Liraglutide showed durable benefits for glycometabolic control and weight management [15]. A recent meta-analysis by Liu et al. showed no association between GLP-1 receptor agonists and the risk of neoplasm development [18]. In randomized controlled trials evaluating liraglutide, no increased risk of neoplasms was identified [18], [19], [20].

Angiosarcomas are often misdiagnosed owing to their rarity and their subtle and varied clinical presentation. In this case, the abdominal wall angiosarcoma was initially presumed to be a large, abscessed hematoma because of the history of repeated liraglutide subcutaneous injections. Despite multiple but because of so rapid recurrences, the working diagnosis of acute hemorrhages remained unchanged. Histological examination of the first specimen was not prioritized and furthermore failed to identify foci of angiosarcoma within the large hematoma partly because of the low clinical suspicion of cancer. Identifying angiosarcoma at the first surgical intervention would have however probably not affected the prognosis, considering the early bulky presentation and the aggressive biology of the disease. Despite adequate surgical resection, angiosarcoma are associated with high recurrence rates [2], [5]. The tumor can rapidly measure upwards to 20 cm or more, and be associated with ulceration, hemorrhage, and edema as it progresses [2].

The most probable risk factor for this patient with primary angiosarcoma of the abdominal wall remains her morbid obesity, and subsequent chronic lymphedema of the abdominal panniculus. The association with liraglutide injection is probably coincidental. It remains probable that the injection itself precipitated the occurrence of a large hematoma within the angiosarcoma. However, considering the rarity of abdominal wall angiosarcomas, a causal relationship between the subcutaneous injections of GLP-1 receptor agonist, must be considered and cannot be excluded.

## 4 Conclusions

Angiosarcoma is a rare and highly aggressive cancer that occasionally originates in the abdominal wall. The majority of cases are reported in patients with morbid obesity. In this described patient's case, in addition to the underlying obesity, a possible association of her liraglutide subcutaneous injections cannot be excluded. In the future, if other cases of abdominal walls angiosarcomas associated with liraglutide subcutaneous injections were to be reported, a possible causality should be further investigated.

## Funding

No funding to declare.

## Ethical approval

Not requested by the Ethic Committee.

## Consent

There is no possibility to identify the patient in the text or in the study material.

## Author contribution

EB, and JN reviewed the case. MD reviewed histopathologic material. XVD was involved in the provision of study material and reviewed imaging. CV provided medical expertise. EB wrote the manuscript. JN reviewed the English syntax. All authors critically reviewed and approved the final version of the article.

## Registration of research studies

Not applicable.

## Guarantor

EB accepts the responsibility for the work.

## Declaration of competing interest

No conflict of interest to declare.
